# Induced Pluripotent Stem Cells Reduce Progression of Experimental Chronic Kidney Disease but Develop Wilms' Tumors

**DOI:** 10.1155/2017/7428316

**Published:** 2017-08-03

**Authors:** Heloisa Cristina Caldas, Fernando Henrique Lojudice, Cinthia Dias, Ida Maria Maximina Fernandes-Charpiot, Maria Alice Sperto Ferreira Baptista, Rosa Sayoko Kawasaki-Oyama, Mari Cleide Sogayar, Christina Maeda Takiya, Mario Abbud-Filho

**Affiliations:** ^1^Laboratory of Immunology and Experimental Transplantation (LITEX), Department of Medicine FAMERP Medical School, Sao Jose do Rio Preto, SP, Brazil; ^2^Department of Medicine/Nephrology-Hospital de Base, FAMERP/FUNFARME, Sao Jose do Rio Preto, SP, Brazil; ^3^NUCEL-NETCEM Cell and Molecular Therapy Center, Medical Clinics Department, School of Medicine, University of São Paulo, Sao Paulo, SP, Brazil; ^4^Genetics and Molecular Biology Research Unit Laboratory-UPGEM, FAMERP Medical School, Sao Jose do Rio Preto, SP, Brazil; ^5^Laboratório de Imunopatologia, Instituto de Biofísica Carlos Chagas Filho, Rio de Janeiro, RJ, Brazil; ^6^Urology and Nephrology Institute, Sao Jose Rio Preto, SP, Brazil

## Abstract

The therapeutic effect of induced pluripotent stem cells (iPSs) on the progression of chronic kidney disease (CKD) has not yet been demonstrated. In this study, we sought to assess whether treatment with iPSs retards progression of CKD when compared with bone marrow mesenchymal stem cells (BMSCs). Untreated 5/6 nephrectomized rats were compared with CKD animals receiving BMSCs or iPSs. Renal function, histology, immunohistochemistry, and gene expression were studied. Implanted iPSs were tracked by the SRY gene expression analysis. Both treatments minimized elevation in serum creatinine, significantly improved clearance, and slowed down progression of disease. The proteinuria was reduced only in the iPS group. Both treatments reduced glomerulosclerosis, iPSs decreased macrophage infiltration, and TGF-*β* was reduced in kidneys from the BMSC group. Both types of treatments increased VEGF gene expression, TGF-*β* was upregulated only in the iPS group, and IL-10 had low expression in both groups. The SRY gene was found in 5/8 rats treated with iPSs. These 5 animals presented tumors with histology and cells highly staining positive for PCNA and Wilms' tumor protein antibody characteristics of Wilms' tumor. These results suggest that iPSs may be efficient to retard progression of CKD but carry the risk of Wilms' tumor development.

## 1. Introduction

Treatments available for chronic kidney disease (CKD), dialysis, and renal transplantation have many drawbacks [1]. We previously showed that rats with CKD treated with bone *marrow-derived* mesenchymal stem cells (BMSCs) injected into the renal parenchyma did stabilize the progression of disease [2]. Despite BMSCs having the capacity for site-specific differentiation into various tissue types, they are limited by the low number of cells available in the injured site; therefore, the perspective of utilizing other stem cells has attracted substantial interest [3].

Pluripotent stem cells have driven attention to approaches aiming to treat some human diseases, and their potential in regenerative nephrology comprises a spectrum from repairing the chronically damaged kidney, at its different stages of the disease, to the establishment of a new functional whole kidney [4].

Embryonic stem cells (ESCs) are pluripotent, have the potential to be self-renewing, and can differentiate into tissues derived from the three germ layers. However, the major obstacle to their use in clinical practice is associated with controversial ethical dilemmas and with uncontrolled growth and cancer formation [5]. Recently, it was demonstrated that induced pluripotent stem (iPS) cells are reprogrammed from fibroblasts by ectopically expressing factors known to be highly expressed in murine ESCs [6–9]. However, during reprogramming, the properties of self-renewal along with unlimited proliferation may cause critical alterations of the transcriptional program and interfere with carcinogenesis. Additionally, there is a concern that virally established cell lines might also lead to the development of tumors [10].

Although iPSs have been proposed to treat some diseases, their therapeutic effect has never been tested on CKD, thus its effect remains unknown.

The objective of the present study was to evaluate iPS efficacy in retarding the progression of CKD.

## 2. Materials and Methods

### 2.1. Isolation and Characterization of Bone Marrow Mesenchymal Cells

BMSCs were isolated from the femurs and tibiae of male Wistar rats. After bone marrow cells were collected by flushing, nucleated cells were isolated with a density-gradient Ficoll-Hypaque (Gibco) and resuspended in Dulbecco's modified Eagle's medium (DMEM) (Gibco) supplemented with 1% penicillin-streptomycin (Invitrogen, Carlsbad, CA, USA) and 10% fetal bovine serum (FBS; Cultilab, Campinas, Brazil). Cells were incubated (37°C in 5% CO_2_) for 14 days as a primary culture. BMSCs were recovered by taking advantage of their tendency to adhere tightly to plastic, and the nonadherent cells were removed by washing. Flow cytometry analyses (FACS Canto; Becton Dickinson, East Rutherford, NJ, USA) were performed for CD31, CD44, CD90, CD45, and CD34 (Caltag Laboratories, Carlsbad, CA, USA), and we tested their potential for adipogenic and osteogenic differentiation, as previously described (data not shown) [2, 11].

### 2.2. Derivation and Characterization of iPSs

Lentivirus were produced by cotransfection in 293T cells of the four packing plasmids (VSV-G, REV, TAT, and HGPM-2) together with the STEMCCA vectors (OKSM or OKS-dSRED) kindly given to us from Professor Gustavo Mostoslavsky. Supernatants were collected every 12 hours, starting 24 hours after transfection, and viral particles were concentrated by centrifugation at 21000*g* for 4 hours at 4°C. Fibroblasts were isolated from Wistar rat skin and grown in DMEM supplemented with 10% fetal calf serum and antibiotics. For transduction, 10^5^ fibroblasts were harvested and incubated for 24 hours with 60 *μ*L of concentrated virus in the presence of polybrene (10 *μ*g/mL) in 35 mm culture plates. After 48 hours posttransduction, the cells were harvested and seeded on irradiated MEFs with rodent ESC medium: DMEM supplemented with 15% FBS (ES-Qualified, Invitrogen), 2 mM L-glutamine, 1X MEM nonessential amino acids (NEAA), 0,1 mM *β*-Mercaptoethanol, 1000 U/mL leukemia inhibitory factor, and antibiotics. The medium was renewed every other day until the iPS colonies appeared (approximately 20–25 days). iPS colonies were first manually picked and then expanded with trypsin [12].

For intracellular staining, iPS colonies were fixed and permeabilized using the 4% paraformaldehyde and 1% Triton X-100. Samples were blocked with 5% BSA for 1 hour at room temperature. iPS colonies were stained overnight with unconjugated anti-OCT4A rabbit mAb (Cell Signaling Technology, 1 : 100) and anti-SOX17 rabbit antibody (Millipore, 1 : 100). The next day, secondary antibody staining with Alexa Fluor 488 (Life Technologies, Carlsbad, CA, USA, 1 : 2000) was performed on iPS colonies that were previously stained with unconjugated antibodies. Subsequently, all iPS colonies were also stained with 49,6-diamidino-2-phenylindole-DAPI (Sigma) before fluorescent microscopic analysis [13].

RNA isolation and real-time PCR assays: total RNA was isolated by RNeasy plus mini kit (Qiagen, Hilden, Germany). cDNA was produced from 2 mg total RNA using reverse-transcribed with random hexamer oligonucleotides using SuperScript III (Invitrogen), according to the manufacturer's protocol. PCR with Taq DNA polymerase (Thermo Scientific, Waltham, MA, USA) was performed using the oligonucleotides listed in supplemental Table S1 available online at https://doi.org/10.1155/2017/7428316. Thermal cycling conditions were as follows: 94°C, 2 minutes; 30 cycles of 94°C for 30 seconds, 58°C for 30 seconds, and 72°C for 1 minute; and then final elongation of 72°C for 5 minutes.

### 2.3. In Vivo Experiments

#### 2.3.1. Ethics Statement

This study was carried out in strict accordance with the recommendations in the Guide for the Care and Use of Laboratory Animals of the National Institutes of Health. The protocol was approved by the Committee on the Ethics of FAMERP Medical School (Sao Jose do Rio Preto, Brazil) (Permit number: 3310/2008). All surgeries were performed under anesthesia, and all efforts were made to minimize suffering. Overdose anesthetic (sodium pentobarbital) was the method of euthanasia in this study.

### 2.4. Animals and Experimental Protocols

A reduction of 5/6 renal mass reduction was used for this study. The 5/6 renal mass reduction model that mimics the structural and functional damage of the CKD procedure was performed as previously described [14]. All animals were provided standard rat chow and water ad libitum.

Briefly, female rats were anaesthetized with ketamine hydrochloride (50 mg/kg) and xylazine (10 mg/kg). Trichotomy and antisepsis of the ventral abdominal area were performed, and the rats were placed in the horizontal dorsal decubitus position. Subtotal renal ablation was performed in a single surgical procedure by subcapsular removal of the right kidney and selective infarction of two thirds of the left kidney by ligation of two of the anterior extrarenal branches of the renal artery, to leave approximately one-sixth of the total kidney tissue mass. Renal ablation was then accomplished through a right nephrectomy and selective ligation of extrarenal branches of the left renal artery in a manner that left approximately 17% of the kidney without lesions.

After surgery female adult rats (*n* = 23) were divided into four groups that underwent intrarenal parenchymal single injections of 0.5 × 10^6^ cells diluted with 10 *μ*L of medium: (i) CKD rats injected in the renal parenchymal with medium (control *n* = 5); (ii) animals treated with BMSCs in the renal parenchyma (*n* = 5); (iii) iPS animals treated with iPSs (*n* = 8); and (iv) sham-operated animals (*n* = 5). All rats were injected with the correspondent treatment immediately after surgery. Rats were euthanized 60 days after surgery, and the kidneys were processed for molecular, histological, and immunohistochemistry analysis.

### 2.5. Renal Function and Blood Pressure Evaluations

Renal function was assessed through measurements of creatinine (SCr), creatinine clearance (CCr), and 24 h proteinuria (PT-24 h) at baseline and 60 days after surgery. CKD progression was measured by the rate of decline in the CCr (RCCr; mL/min/day). At the end of 60 days, the animals were weighed and sacrificed, and histological examinations were performed in the remnant kidneys.

Plasma and urine SCr concentrations were determined using a colorimetric assay (Jaffe reaction, spectrophotometer BTS 310; Biosystems S.A., Barcelona, Spain). Creatinine clearance was calculated from measurements of serum and urinary SCr levels. Blood pressure was assessed by an indirect tail-cuff method (Insight LTDA, Ribeirão Preto, SP, Brazil), and the mean of four measurements was used for analysis.

Animals were placed in metabolic cages (Tecniplast, Buguggiate-VA, Italy) to collect urine (24-hour samples).

### 2.6. Histological and Immunohistochemical Analysis

For light microscopy, tissues were fixed in neutral formalin and embedded in paraffin, and 3 *μ*m sections were stained with hematoxylin and eosin (H&E) and Masson's trichrome. A semiquantitative score was used to determine the extent of glomerulosclerosis and tubulointerstitial changes, as described previously [15]. In brief, a glomerular injury score was obtained by multiplying the severity of damage (0 to 4+) by the percentage of glomeruli with that degree of injury. For tubulointerstitial injury (tubular dilatation, atrophy, interstitial edema, fibrosis, and mononuclear cell infiltrates), a score was assigned according to the appropriate proportion of tissue affected (0 to 4+). All sections were evaluated by an observer who was blinded to the experimental protocol.

Immunohistochemical procedures were performed on 5 *μ*m-thick paraffin-embedded kidney sections using the following antibodies: mouse monoclonal antibody against proliferating cell nuclear antigen (PCNA) (Dako, Carpinteria, CA, USA, 1 : 200), (macrophages) CD68 (Serotec, Oxford, UK, 1 : 250), VEGF (Ab1316—Abcam Cambridge, UK, 1 : 400), TGF-*β* (Ab80436—Abcam, Cambridge, UK, 1 : 250), mouse monoclonal antibody against *α*-smooth muscle actin (*α*-sma) (clone 1A4, cat. M0851, 1 : 100), rabbit monoclonal antibody to vimentin (clones V9, cat. M3200, Spring, USA, 1 : 100), and a rabbit polyclonal against WT1 antibody (C-19, sc-192, Santa Cruz Biotechnologies, Dallas, TX, USA, 1 : 250). After dewaxing and rehydrating, sections were permeabilized with a 0.5% Triton-X-100 in phosphate-buffered saline (PBS) pH 7.4 and submitted to endogenous peroxidase inhibition (15 minutes with 3% H_2_O_2_ in methanol). Heat-mediated antigen retrieval in microwave was performed (5 minutes) using either citrate buffer, pH 6.0, or Tris-EDTA buffer, pH 9.0, at 800 kW, the former for vimentin, *α*-sma, and PCNA, and the latter for WT1. After cooling the histological sections, blocking of the nonspecific binding of immunoglobulin to the tissue was performed, and the primary antibodies were then incubated for about 16 hours at 4°C in a humidified chamber. The sections were washed in a 0.25% Tween-PBS solution, and then the secondary antibodies were incubated (Nichirei-Histofine® Simple Stain Rat MAX-PO (mouse) and Simple Stain Rat MAX-PO (rabbit), both for rat tissue, cat. 414171 and cat. 41434, resp.). The chromogen substrate was diaminobenzidine (Liquid DAB, Dako, cat. number K3468). Negative control slides were incubated with mouse or rabbit isotype immunoglobulins or with the antibody diluents solution.

### 2.7. RNA Extraction and Quantitative Real-Time PCR Analysis

Total RNA was isolated from the kidney using Trizol Reagent (Life Technologies), and RNA concentration was determined using a Qubit fluorometer according to the manufacturer's protocol (Invitrogen, Carlsbad, CA, USA). Reverse transcriptase reactions were performed with high-capacity RNA-to-CDNA kit (Applied Biosystems, Foster City, CA, USA) using 1 *μ*g of total RNA.

QPCR was performed using the StepONE plus real-time PCR system (Applied Biosystems) using TaqMan probes (Applied Biosystems) for glyceraldehyde-3-phosphate dehydrogenase (GAPDH) (Rn99999916_s1; endogenous control gene), IL-6 (Rn00561420_m1), IL-10 (Rn00563409_m1), VEGF (Rn00582935_m1), and TGF-*β* (Rn00572010_m1), according to the manufacturer's recommendations.

Values are expressed relatively to the RNA obtained from the untreated groups (Sham). The quantification of the target genes were normalized by the endogenous control gene. The threshold cycle (Ct) for the target gene and the Ct for the internal control were determined for each sample, run in duplicates. The relative expression of mRNA was calculated by the 2^−ΔΔCT^ method [16].

### 2.8. Presence of Gene SRY in Renal Tissue

BMSCs and iPS were isolated from male rats and injected into female rats, enabling detection of the SRY gene (located on the Y chromosome ([GenBank: FJ168067.1])) as an index of engraftment. Total RNA was isolated from the kidney using a Trizol Reagent (Life Technologies), and RNA concentration was determined using Qubit fluorometer according to the manufacturer's protocol (Invitrogen, Carlsbad, CA, USA). Reverse transcriptase reactions were performed with a high-capacity RNA-to-cDNA kit (Applied Biosystems) using 1 *μ*g of total RNA. qPCR was performed using the StepONE plus real-time PCR system (Applied Biosystems) and TaqMan probes (Applied Biosystems) for GAPDH (Rn99999916_s1; endogenous control gene) and *SRY* (Rn04224592_u1). The real-time PCR conditions consisted of an initial denaturation step of 10 minutes at 95°C, followed by 40 cycles at 95°C for 15 seconds, and at 58°C, for 1 minute.

### 2.9. Statistical Analysis

Analyses were performed using StatsDirect version 3.0 (*StatsDirect* Ltd, Cheshire, UK), with the critical level set at *p* < 0.05. Data were expressed as mean ± standard deviation. Comparisons among multiple groups were performed using analyses of variance (ANOVA). When *p* values were significant, differences between the groups were specified with Tukey's multiple comparison posttests. When comparing data, a two-sided Student's *t*-test and Mann–Whitney *U* test were used.

## 3. Results

### 3.1. Generation and Characterization of BMSC and iPS In Vitro

Bone marrow-derived BMSCs from rats which showed stable fibroblast-like phenotypes in culture were isolated by adherence separation. The BMSCs were able to differentiate into adipocytes and osteoblasts and expressed the common markers of BMSCs.

**Figure 1:**
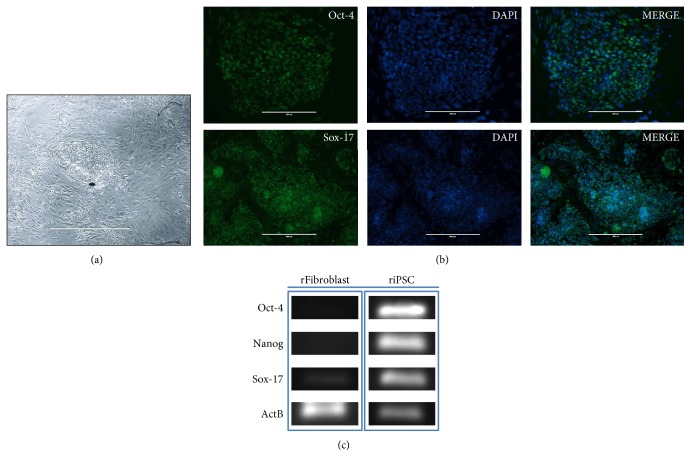
Characterization of induced pluripotent stem cells (iPSs). (a) Morphology of the iPS cell colonies. (b) Immunofluorescent staining of rat-induced pluripotent stem cells for the expression of the pluripotency markers OCT-4 (upper panels, scale bar 400 *μ*m) and SOX-17 (lower panels, scale bar 1000 *μ*m) and overlay with the control staining of the nucleus with DAPI (Sigma). (c) RT-PCR analysis of gene expression in undifferentiated riPS cells.

Rat iPSs were generated from skin fibroblasts using STEMCCA (OKSM) and formed colonies that were clonally expanded and displayed the typical morphology of ESC colonies (Figure 1(a)).

Immunostaining showed that transduced iPSs expressed pluripotent markers OCT-4 and SOX-17 (Figure 1(b)). To test the pluripotent gene expression during iPS differentiation, RT-PCR analysis was performed (Figure 1(c)).

### 3.2. Renal Functional Studies

Table 1 depicts the effect of BMSC and iPS therapies on the renal function of rats undergoing 5/6 nephrectomy. After 60 days, untreated CKD animals doubled their SCr, whereas those treated with BMSCs or iPSs had increases of 35% and 57%, respectively (*p* = NS versus sham group). Both types of cells increased by twofold the creatinine clearance (*p* < 0.05 versus CKD) and slowed down the progression of disease (RCCr) during the period of observation, although iPS did not reach statistical significance compared with CKD. The 24-hour proteinuria was significantly reduced only in the iPS group. Mean arterial blood pressure was not affected by treatments.

### 3.3. Histopathological and Immunohistochemistry Analysis

By the end of the study, all CKD rats showed significant reduction in glomerulosclerosis with both treatments, but the tubulointerstitial damage remained unchanged at light microscopy. Overall, BMSC- and iPS-treated animals seemed to have less severe CKD throughout the remnant kidney. The kidney chronicity scores of the three groups are compared in Table 2.

**Figure 2:**
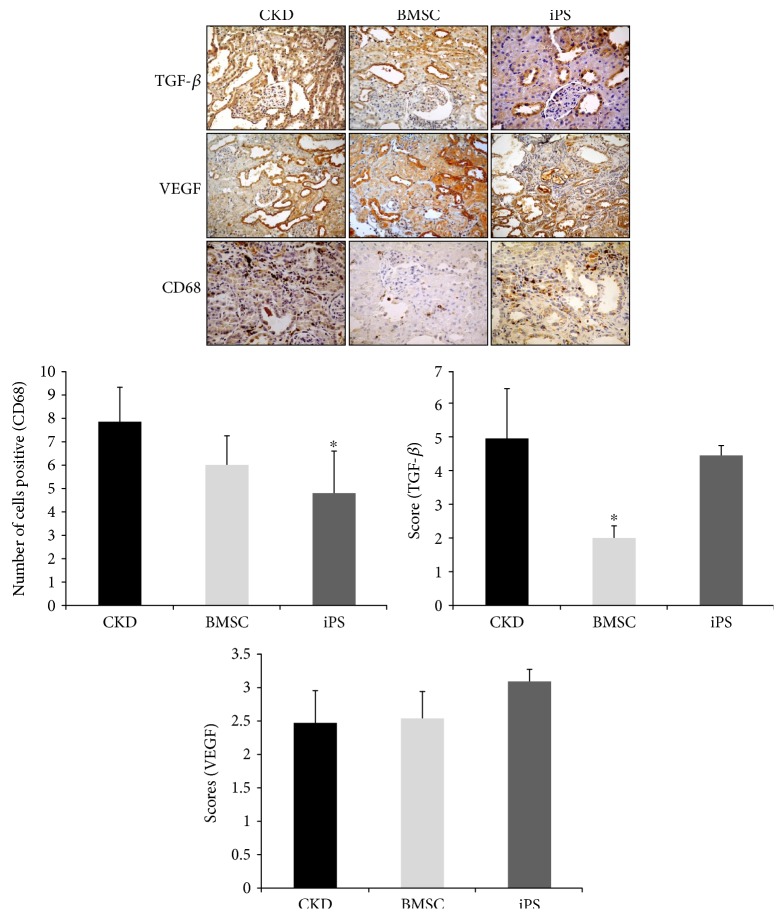
Immunohistochemistry of renal tissue from 5/6 nephrectomized rats, BMSC and iPS. Immunolocalization of CD68-positive cells; staining for VEGF; expression of TGF-*β* in the renal cortex. Data are expressed as means ± SD. Representative images of immunolocalization of TGF-*β*, VEGF, and CD68 cells (macrophages) in the renal cortices of the CKD, BMSC, and iPS groups on postoperative day 60 (magnification: 400x). ^∗^*p* < 0.05 versus CKD.

When compared with the CKD animals, the immunohistochemistry showed significantly less macrophages CD68+ infiltration in the kidneys of iPS-treated rats, whereas lower TGF-*β* was observed in the BMSC group. Although they did not reach statistical significance, a trend toward to an increase in the expression of VEGF was observed in kidneys receiving iPS treatment (Figure 2).

### 3.4. iPS Treatment and Wilms' Tumor (Nephroblastoma) Formation

Macroscopic examination of kidneys from the iPS group revealed tumor-like formations in 5 out of 8 remnant kidneys. No kidneys from other groups presented tumors.

**Figure 3:**
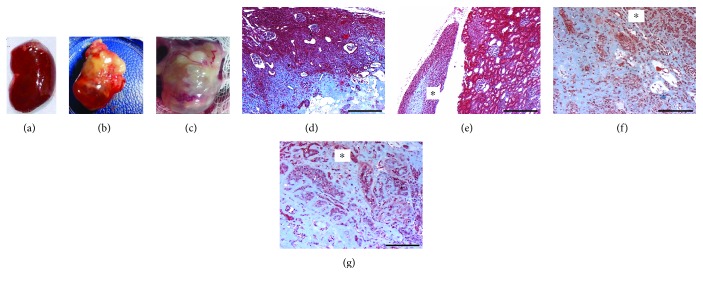
The macroscopic and microscopic image shows tumors formed by induced pluripotent stem cells (iPSs) infused into the renal parenchyma after 60 days. (a) Kidney from wild-type rat shows normal size and appearing, while (b) and (c) represent macroscopic image of the cut surface of the kidney tumor. (d) Photomicrography of a chronic injured kidney showing loss of the renal architecture as a result of a high cellularity in the renal interstitium. Masson-trichrome staining. Bar: 500 *μ*m; (e) perirenal tissues infiltrated with tumoral cells (∗) Masson-trichrome staining. Bar: 500 *μ*m. (f, g) Photomicrography of the tumoral area showing tumor cells surrounded by extracellular matrix (ECM) (∗) and organized in rows or nests resembling blastemal tissue (∗). Masson-trichrome staining. Bar: 200 *μ*m.

**Figure 4:**
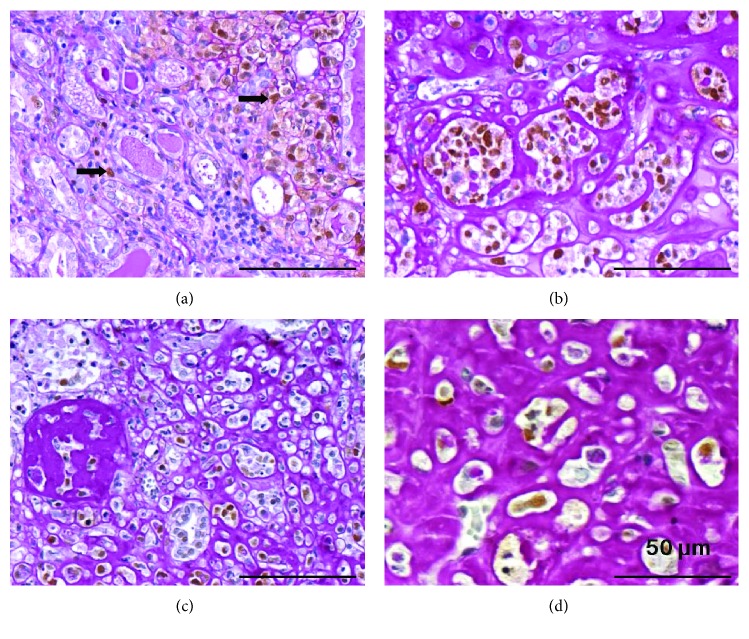
(a) Tumor cells infiltrating the renal interstitium with PCNA-positive nuclei. Notice that the majority of tumor cells were stained in brown by the PCNA antibody. Immunoperoxidase reaction using PCNA antibody revealed with the chromogen 3,3′-diaminobenzidine (DAB) in brown, followed by periodic acid-Schiff (PAS) staining. Bar: 100 *μ*m. (b) Tumor cells arranged as either isolated or grouped cells surrounded by a dense PAS reactive extracellular matrix, some with nuclei positive for PCNA antibody. PCNA immunoperoxidase reaction revealed with DAB and followed by PAS staining. Bar: 100 *μ*m. (c) In an area of the tumor, the presence of a nodular structure composed of PAS-positive ECM. PCNA immunohistochemistry revealed with DAB and followed by PAS staining. Bar: 100 *μ*m. (d) Tumor area composed by a dense PAS+ ECM, with tumor cells immersed resembling cartilaginous tissue. PCNA immunohistochemistry revealed with DAB and followed by PAS staining. Bar: 50 *μ*m.

**Figure 5:**
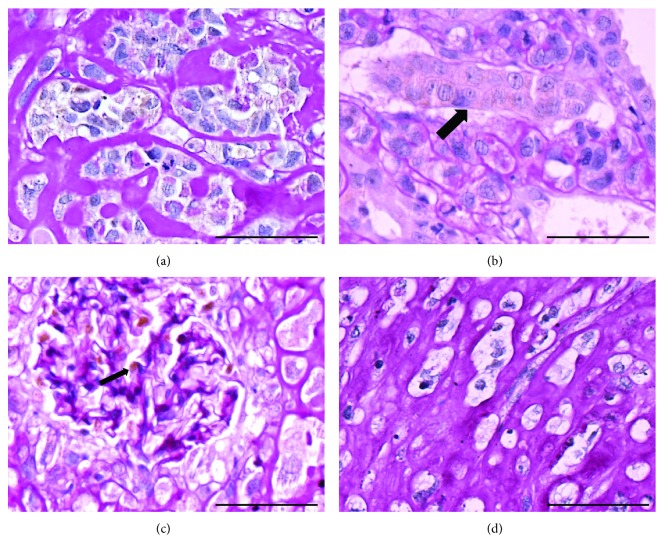
(a) Nests of tumor composed with anaplastic cells with a light, heterogeneous cytoplasmic staining for WT1 antibody. WT1 immunohistochemistry revealed with diaminobenzidine (DAB) and followed by periodic acid-Schiff (PAS) staining. Bar: 50 *μ*m. (b) Tumor cells surrounding a renal tubule. Notice the more homogeneous WT1 staining of the cytoplasm of the renal tubule (→) contrasting with the lightly reactive tumor cells. WT1 immunoperoxidase reaction revealed with DAB and followed by PAS staining. Bar: 50 *μ*m. (c) Internal positive control of the reaction. Glomerular podocytes WT1 positive in histological section of chronic renal injured kidney treated with iPSs. Bar: 50 *μ*m. (d) Negative control of the reaction. Histological section of an iPS-treated animal incubated with nonimmune rabbit immunoglobulin instead of the primary antibody. Bar: 50 *μ*m.

Histopathological analysis revealed tumors with characteristics of nephroblastoma, presenting the classical triphasic histology, comprising the blastemal tissue, hyperchromatic nuclei cells with scanty cytoplasm, primitive tubules, and glomeruli formation admixed with mesenchymal and epithelial cells (Figure 3). Tumor cells infiltrating renal interstitium had nuclei stained positive for PCNA antibody (Figure 4), were arranged either isolated or grouped, and in some areas resembled cartilaginous tissue surrounded by dense periodic acid-Schiff (PAS) reactive extracellular matrix (Figure 4). Tumor cells also had heterogeneous cytoplasmic staining positive for WT1 (Figure 5) and negative for vimentin and *α*-sma antibodies (data not shown).

### 3.5. Presence of SRY Gene and Wilms' Tumor

To track the transplanted cells, we searched for SRY gene sequences of the Y chromosome from the donor rat male cells into the remnant kidney tissue of the female recipient rats. The SRY gene was found in 5/8 (62.5%) rats from the group treated with iPSs and, coincidentally, all 5 animals had nephroblastomas. We could not find the Y chromosome in any other animal in any other groups. Because the iPS-treated group had lost more weight after 60 days, we also speculate that tumors could have negatively impaired the renal function of those kidneys. Then, we studied only the animals without tumors (*n* = 3) and compared with those with tumors (*n* = 5) and studied separately the renal function of both subgroups. There was a greater weight loss in the rats with tumors, but to our surprise, the renal function parameters were not different between the two subgroups and were similar to the results observed in the whole iPS group (data not shown).

### 3.6. Gene Expression of Cytokines in the Renal Tissue

**Figure 6:**
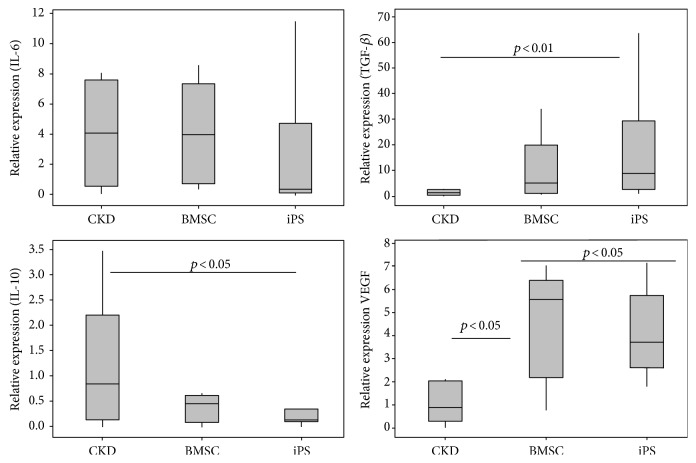
Cytokine expression at the kidney tissue. Gene expressions in the kidneys of treated and nontreated (CKD) animals, generated by referencing each gene to GAPDH as an internal control. Data expressed as mean of 2^−ΔΔCT^ ± DP.

A significant increase in VEGF gene expression was observed in both the BMSC- and iPS-treated groups when compared to the untreated CKD animals (Figure 6). Similarly, TGF-*β* was significantly more expressed in the iPS group than in the CKD group (*p* < 0.01) although numerically, the relative mean expression of TGF-*β* transcripts was twofold greater in IPSs than in BMSCs. IL-10 gene expression was significantly decreased in iPS-treated animals (*p* < 0.05 versus CKD), and no differences were found with regard to IL-6 gene expression.

## 4. Discussion

Due to their plasticity, BMSCs derived from different sources have been successfully used in various strategies to treat experimental acute and chronic kidney disease [17, 18]. However, because of the need for invasive procedures to obtain the cells, variations in the quantity and quality of isolated cells, the low frequency found in bone marrow aspirates, and controversial issues regarding their differentiation into nonmesodermal tissues, their routine use in the clinical setting remains limited [17, 18].

Development of the iPS technology circumvented some BMSC hurdles and opened new promising therapeutic possibilities to produce cells suitable for use in regenerative therapy.

In the present study, we generated iPSs from rat skin fibroblasts and compared their functional effects with those of BMSCs on the progression of the experimental CKD. Our data suggest that both cells, BMSCs and iPSs, were effective in improving renal function, because they increased the CCr, attenuated the elevation in SCr, and slowed down the RCCr at the end of the study period (Table 1). The significant reduction in the PT24 observed only in the iPS-treated group could be related to yet unknown mechanisms by which iPSs can be differentiated into mature podocytes, resulting in decreased podocyte permeability and less proteinuria [19].

Because the renal function of rats treated with BMSCs fared slightly better than rats treated with iPSs, we thought that the five tumors arousing in the iPS-remnant kidneys could have compromised the renal function of those rats. However, individual analysis of subgroups with and without tumors did not show any differences with regard to renal function, although we do acknowledge that the remaining number of animals in each group does not allow drawing definitive conclusions.

The finding of Wilms' tumor-like was unexpected, because teratomas have been reported associated with iPS. However, teratomas are a benign tumors constituted by a mixture of differentiated tissues and organotypic structures derived from the three germ layers, such as the teeth and skin with its appendages (hair and sebaceous glands) [20, 21]. The malignant transformation of teratoma, the teratocarcinoma, contains embryonal carcinoma cells. The embryonal carcinoma cells are pluripotent, capable of self-renewal, originate repeatedly transplantable tumors, and therefore represent specific tumor stem cells [22–24]. The malignant transformation of a teratoma consists of the transformation of a somatic teratomatous component of a germ cell tumor to a nongerm cell tumor malignant phenotype. The most frequent histologic subtypes of the malignant counterpart are the rhabdomyosarcoma, adenocarcinoma, and primitive neuroectodermal tumors [25].

In our animals injected with iPS, the developed tumor is without doubt a malignant one since it is extremely anaplastic, with a high proliferation index, and is invasive as it is seen between renal structures (tubules or glomeruli) at the renal capsule and perirenal adipose tissue (Figures 5(a) and 5(b)). Histologically, it is constituted by malignant cells forming aggregates like rows or glands (Figure 5(b)), with features of blastemal tissue (Figure 4(d)). The stroma is loose (mixoid) or dense, PAS positive. Therefore, the histological features are very similar to those seen in Wilms' tumor or nephroblastoma, an embryonal type of renal cancer, the most common solid malignant neoplasms in children.

Wilms' tumor imitates the classic triphasic aspect of the embryonic kidney histology (blastema, epithelia, and stroma), the most characteristic pattern. However, biphasic and monophasic aspects are often observed. The majority of Wilms' tumor components represent stages of normal or abnormal nephrogenesis, and nonrenal elements including skeletal muscle, bone, and cartilage can be present [26]. The blastemal component is characterized by densely packed oval to spindle undifferentiated cells. The epithelial component is constituted by abortive tubular and glomerulus-like structures. The stroma is formed by differentiated or less differentiated nonepithelial cells, occasionally presenting differentiation towards striated muscle, bone, and cartilage. Although not necessary for the histopathological diagnosis, the immunoprofile of Wilms' tumor shows that blastemal cells regularly express vimentin and may also show positivity for neuron-specific enolase, desmin, and cytokeratin [27, 28].

The WT-1 gene product is not present in all nephroblastomas and may be present in various other tumors. In nephroblastomas, WT1 is nuclear and correlates with tumor histology. Low levels or absence of WT1 is confined to areas of stromal differentiation and terminal epithelial differentiation, whereas high levels of WT1 are seen in areas of blastemal and early epithelial differentiation [29, 30]. Moreover, the blastemal compartment represents the neoplastic analogue of the metanephric mesenchyme in development and contains multiple undifferentiated and differentiated cell types suggestive of a multipotent cell of origin [31]. Therefore, WT is considered as a prototype of differentiation failure in human cancer, since it shares the histology of the fetal kidney, including blastema, stroma, and differentiating tubular epithelium [31–34].

Wilms' tumor and Wilms' tumor stem-like xenografts overexpress regulatory genes of the stem/progenitor cell pool corresponding to the earliest stages of normal human kidney development [33]. Besides that, evidences demonstrate in WT the presence of epigenetic alteration in several embryonic stem cell genes [35, 36].

During kidney development, the blastema usually disappears by 36 weeks gestation. However, approximately 1% of infants at birth retain residual blastemal within their kidney [37, 38]. The nephrogenic rest is considered as a focus of abnormally persistent nephrogenic cells, which can be induced to form a Wilms' tumor [37].

IPS-induced teratomas and other types of malignant tumors could result from the self-renewal capacity and pluripotency of these cells, probably because reprogramming the unlimited proliferation and global alterations of the transcriptional program could initiate carcinogenesis [39, 40]. Ohnishi et al. showed that premature termination of reprogramming could lead to WT formation, and they suggested that, in vivo, the WT could be the result of incomplete reprogramming [41]. Although the oncogenesis may be circumvented with techniques allowing the establishment of iPSs without transfection of the oncogene c-Myc, the tumor formation induced by the iPSs remains a matter of concern [42].

Previous studies have previously showed the beneficial effect of BMSCs in a chronically damaged kidney by reducing the inflammation and fibrosis process. However, the mechanisms underlying the improvement of renal function are much more complex and involve paracrine and endocrine actions, secretion of growth factors, anti-inflammatory cytokines, antiapoptotic factors, antifibrogenic factors, and angiogenic factors, all acting together to build an appropriate microenvironment allowing the reduction of injury and fibrosis and promoting repair [43–48].

Corroborating this thought, our study shows that the histology of remnant kidneys had significantly less glomerulosclerosis in both treated groups, although chronicity of the tubulointerstitial compartment remained unchanged. Immunohistochemistry of renal tissue revealed different patterns for the two types of cells, with BMSCs showing reduction in TGF-*β* and iPSs exhibiting much less kidney macrophage infiltration, both findings suggesting an anti-inflammatory response leading to less fibrosis.

Although we found discrepancies between protein level and gene expression with regard to VEGF in the three groups of animals, both therapies presented very similar renal transcript profiling showing high expression of VEGF and TGF-*β* and low IL-10 gene expression, which suggests the involvement of these molecules in the ongoing regenerative process or a tumor-induced response. While the VEGF is a renoprotective factor required for the glomerular hypertrophy and endothelial cell proliferation in response to nephron reduction, the TGF-*β* activation signals may suggest the induction of renal progenitors (from the intermediate mesoderm) [49].

In an attempt to track the administered iPS cells into the kidney, we tried to detect the SRY gene from implanted donor cells. Interestingly, we observed that the SRY gene was found exclusively in the five remnant kidneys exhibiting WT rising questions whether the iPSs used as treatment may have acted as tumor-initiating cells leading to the WT formation.

There is no doubt that the association of iPSs with WT tumors deserves caution when extrapolated to the clinical application. However, our data may also suggest that because the histology of WT is similar to that of fetal kidneys and the WT1 transcription factor expression may be required for kidney development and normal organ function and considering that WT cells may share chromatin profiles intimately connected to kidney organogenesis, a link between the use of iPSs and kidney regeneration could be possible and therefore a promising treatment for CKD [42, 50].

We acknowledged some limitations in this study such as the controversies with regard to the ideal place to administer the cell therapy and the small numbers of animals studied.

In conclusion, our results using iPSs as cell therapy for the CKD experiments are encouraging, but caution is still required regarding the potential for WT formation.

## Supplementary Material

The Sequences for the Primers of Reverse Transcription-Polymerase Chain Reaction.

## Figures and Tables

**Table 1:** Renal function parameter measurements at the end of the study (day 60).

Parameters	Groups			
	Sham	CKD	BMSC	iPS
SCr (mg/dL)	0.6 ± 0.1^a^	1.2 ± 0.2	0.81 ± 0.13	0.94 ± 0.37
PT24h (mg/24 h)	1 ± 0.2^b,c,d^	76.6 ± 41.5^f^	57.12 ± 22^h^	12.2 ± 42.6
CCr (mL/min)	0.75 ± 0.1^e^	0.31 ± 0.04	0.66 ± 0.2^i^	0.60 ± 0.2^j^
RCCr (mL/min/d)	0.0001 ± 0.006	0.012 ± 0.002^g^	0.007 ± 0.004	0.009 ± 0.006
Mean arterial pressure (mmHg)	127 ± 1	192 ± 35.7	178 ± 54.5	176 ± 59
Body weight change (g)	11 ± 48	−17.6 ± 22.2	5.8 ± 30.6	−12 ± 32

Results are mean ± SD. SCr = serum creatinine; RCCr = rate of decline of CCr; PT24h = 24-hour proteinuria; CCr = creatinine clearance; CKD = chronic kidney disease; BMSC = mesenchymal stem cell; iPS = induced pluripotent stem cells. ^a^*p* < 0.01 versus CKD; ^b^*p* < 0.01 versus CKD; ^c^*p* < 0.01 versus BMSC; ^d^*p* < 0.01 versus iPS; ^e^*p* < 0.01 versus CKD; ^f^*p* < 0.05 versus iPS; ^g^*p* < 0.05 versus BMSC; ^h^*p* < 0.05 versus iPS; ^i^*p* < 0.05 versus CKD; ^j^*p* < 0.05 versus CKD.

**Table 2:** Histological changes and the effect of treatment with BMSC and iPS in 5/6 nephrectomized animals.

	CKD	BMSC	iPS
GS	23.6 ± 13^a^	4 ± 3	1.8 ± 1
TID	78.2 ± 15	43 ± 29	62 ± 56

CKD: untreated chronic kidney disease; BMSC: rats treated with mesenchymal stem cells; iPS: rats treated with induced pluripotent stem cells; GS: glomerular sclerosis; TID: tubular damage. Data are expressed as means ± SD (^a^*p* < 0.01 versus BMSC and iPS).
