# Supplementary material for: Induced Pluripotent Stem Cells Reduce Progression of Experimental Chronic Kidney Disease but Develop Wilms' Tumors
Source: Stem Cells Int. 2017 Aug 3;2017:7428316. doi: 10.1155/2017/7428316 (PMC5560097; doi:10.1155/2017/7428316)
Supplement: Supplementary file 1 — The Sequences for the Primers of Reverse Transcription-Polymerase Chain Reaction. [file 7428316.f1.docx]

**Table S1:** The Sequences for the Primers of Reverse Transcription-Polymerase Chain Reaction

| **Gene name** | **Primer Sequence (5` to 3`)** |
| --- | --- |
| Oct-4_F | ATAGATCTCATGGCTGGACACCTGGCT |
| Oct-4_R | AGTCTAGACTCAGTTTGAATGCATGGGAGATGT |
| Nanog_F | CATCCTGAACCTCAGCTACAAACA |
| Nanog_R | TTGCTATTCTTCGGCCAGTTGT |
| Sox-17_F | TTTCATGGTGTGGGCTAAGGA |
| Sox-17_R | GCGCCTTCCACGACTTGC |
| ActB_F | AAGGCCAACCGTGAAAGATG |
| ActB_R | GTGGTACGACCAGAGGCATACA |
